# Bupropion Shows Different Effects on Brain Functional Connectivity in Patients With Internet-Based Gambling Disorder and Internet Gaming Disorder

**DOI:** 10.3389/fpsyt.2018.00130

**Published:** 2018-04-10

**Authors:** Sujin Bae, Ji Sun Hong, Sun Mi Kim, Doug Hyun Han

**Affiliations:** ^1^Industry Academic Cooperation Foundation, Chung-Ang University, Seoul, South Korea; ^2^Department of Psychiatry, College of Medicine, Chung-Ang University, Seoul, South Korea

**Keywords:** Internet gaming disorder, gambling disorder, bupropion, default mode network, cognitive control network

## Abstract

**Introduction:**

Internet gaming disorder (IGD) and gambling disorder (GD) share similar clinical characteristics but show different brain functional connectivity patterns. Bupropion is known to be effective for the treatment of patients with IGD and GD. We hypothesized that bupropion may be effective for the treatment of Internet-based gambling disorder (ibGD) and IGD and that the connections between the default mode network (DMN) and cognitive control network (CCN) would be different between ibGD and IGD patients after 12 weeks of bupropion treatment.

**Methods:**

16 patients with IGD, 15 patients with ibGD, and 15 healthy subjects were recruited in this study. At baseline and after 12 weeks of bupropion treatment, the clinical symptoms of patients with IGD or ibGD were assessed, and brain activity was evaluated using resting state functional magnetic resonance imaging.

**Results:**

After the 12-week bupropion treatment, clinical symptoms, including the severity of IGD or GD, depressive symptoms, attention, and impulsivity improved in both groups. In the IGD group, the functional connectivity (FC) within the posterior DMN as well as the FC between the DMN and the CCN decreased following treatment. Moreover, the FC within the DMN in the IGD group was positively correlated with changes in Young Internet Addiction Scale scores after the bupropion treatment period. In the ibGD group, the FC within the posterior DMN decreased while the FC within the CCN increased after the bupropion treatment period. Moreover, the FC within the CCN in the ibGD group was significantly greater than that in the IGD group.

**Conclusion:**

Bupropion was effective in improving clinical symptoms in patients with IGD and ibGD. However, there were differences in the pharmacodynamics between the two groups. After 12 weeks of bupropion treatment, the FC within the DMN as well as between the DMN and CCN decreased in patients with IGD, whereas the FC within the CCN increased in patients with ibGD.

## Introduction

Internet-based gambling is a modified form of gambling using Internet-enabled devices, including computers, mobile phones, and digital television (1, 2). Due to the characteristics of online systems such as speed and ease of accessibility, internet-based gambling may have a rapid feedback system and provide easy access to variable betting options (1, 2). Over the last two decades, Internet gaming disorder (IGD) has been regarded as a mental disease characterized by the urge for game (gambling) play, extensive playing time, and harmful side effects (3). Due to the similarities between IGD and internet-based gambling disorder (ibGD) with respect to the clinical symptoms of excessive use and the potential adverse effects, several studies have suggested that IGD may be diagnostically similar to ibGD (4). Because of these diagnostic similarities, medications for gambling disorder (GD), including escitalopram and bupropion, have also been applied to IGD (5–8). However, there has been controversy regarding the classification of IGD as an addiction or impulse control disorder (3, 9, 10) as well as the differences in brain functional connectivity (FC) within the cognitive network between the two diseases (11). Therefore, a comparison of the effects of medication on the two diseases is warranted.

Among the several medications known to be effective for reducing the symptoms of GD (5, 6), bupropion has been suggested to improve the symptoms of IGD (8, 12). Bupropion is effective for treating patients with GD by decreasing gambling behavior and the amount of money spent (5, 6). Black et al. (5) reported that bupropion was effective and well tolerated in patients with GD (5). Dannon et al. (6) have suggested that bupropion is as effective as naltrexone based on its mechanism of regulating dopamine release. Bupropion acts to inhibit the reuptake of dopamine and norepinephrine by stimulating acetylcholine, hydroxytryptamine, gamma aminobutyric acid receptor, and endorphin signaling (13). These neurochemical systems may be associated with the urges, craving, and enjoyment accompanying gambling behaviors and addiction to drugs of abuse (14). The opioid antagonist naltrexone may block alcohol-induced dopamine release in the nucleus accumbens, which reduces the craving for alcohol and promotes abstinence (15). Studies have suggested that bupropion could improve the symptoms of IGD by improving comorbid depressive symptoms and inducing changes in brain activity (8, 16). Twelve weeks of bupropion treatment has been shown to improve IGD symptoms as well as depressive symptoms in patients with major depressive disorder and IGD (8). In another study, 6 weeks of bupropion treatment reduced the severity of IGD by decreasing brain activity within the dorsolateral prefrontal cortex in response to game stimulation (16).

In our previous study comparing the brain connectivity of the default mode network (DMN) and cognitive control network (CCN) between IGD and ibGD, both groups showed a similar decrease in FC in the DMN. However, FC within the CCN was increased in the IGD group but not the ibGD group (11). The DMN refers to functionally grouped areas that are synchronously deactivated during task performance and mainly activated during rest (17). The DMN was usually thought to consist of the posterior cingulate cortex (PCC), precuneus, medial frontal cortex (mPFC), ventral anterior cingulate cortex (ACC), and lateral (LP) and inferior parietal lobes (IP) (17). In patients with substance dependence, the brain FC within the DMN was positively correlated with impulsivity (18). In patients with GD, decreased FC within the DMN from the PCC to the left superior frontal gyrus, right middle temporal gyrus, and precuneus was reported. In addition, the severity of GD was negatively correlated with the FC from the seed PCC to precuneus (19). However, previous studies on FC within the DMN in IGD have shown variable findings (11, 12). The FC within the posterior parts of the DMN in patients with IGD was decreased (11). By contrast, FC between the DMN and the salience network was increased in patients with IGD (12).

The CCN is correlated with the process of employing executive functions, including attention, planning, and working memory for guiding appropriate behaviors to achieve specific goals (20). It includes the dorsal regions of the lateral prefrontal cortex (DLPFC), ACC, and parietal cortex (20). As gambling and Internet gaming are associated with goal-directed decision-making (21), several scholars have suggested that the FC within the CCN would be associated with gambling and IGD (22). Moreover, the conflict and uncertainty resulting from risky decision-making during gambling tasks can activate the dorsal prefrontal cortex (23).

We hypothesized that bupropion might be effective for the treatment of ibGD and IGD. However, the mechanism of bupropion action in the treatment of ibGD and IGD in terms of brain connectivity between DMN and CCN would differ. We hypothesized that bupropion would decrease the FC between the DMN and CCN in the IGD group, but would increase the FC within the CCN in the ibGD group.

## Materials and Methods

### Participants

**Figure 1:**
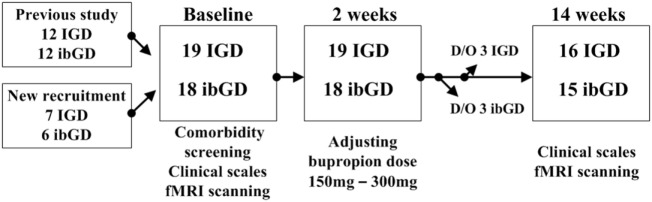
Study procedure. Abbreviations: IGD, Internet gaming disorder; ibGD, Internet-based gambling disorder; D/O, dropped out; fMRI, functional magnetic resonance imaging.

Of the 15 patients with IGD and 14 patients with ibGD who participated in our previous study comparing brain connectivity (11), 12 patients with IGD and 12 patients with ibGD agreed to participate in this study. In addition, seven patients with IGD and six patients with ibGD who visited the outpatient department of OO hospital were newly recruited in this study (Figure 1). All participants were screened with the DSM-IV structural clinical interview for assessing psychiatric comorbidity (24). During the follow-up period, three patients with IGD and three patients with ibGD dropped out because of voluntary termination and changes in medication. Finally, 16 patients with IGD and 15 patients with ibGD completed the study protocol (Figure 1). The inclusion criteria were as follows: (1) diagnosed with IGD based on the DSM-5 or determined to have ibGD. We used the diagnostic criteria of GD and adapted it to form the inclusion criteria for ibGD, but changed “problematic gambling” in the DSM-5 to “ibGD,” (2) adult (>18 years old), (3) male, and (4) psychiatric medication-naïve. The exclusion criteria were as follows: (1) other comorbid medical or psychiatric diseases, (2) low intelligence quotient (IQ) (less than 80), (3) contraindications for MRI scanning such as claustrophobia and metal implantation, and (4) history of substance abuse with the exception of social alcohol drinking and smoking.

### Procedure

At baseline, all participants were asked to complete questionnaires for demographic data and clinical symptoms. The symptom severity of ibGD and IGD was assessed with the Yale-Brown Obsessive Compulsive Scale for pathologic gambling (YBOCS-PG) (25) and Young Internet Addiction Scale (YIAS) scores (26), respectively. Four more clinical symptom assessment scales were applied to all participants: the Beck Depression Inventory (BDI) (27) for depressive mood symptoms, the Korean ADHD Rating Scale (K-ARS) (28) for attention symptoms, and the Behavioral Inhibitory System and Behavioral Activation System scales for inhibitory and excitatory personal traits for aversive or appetitive motivations in behavior (29). The IQ of all participants was assessed using the Korean-Wechsler Adult Intelligence Scale (30). In addition, all participants were scanned to analyze brain FC *via* resting state functional magnetic resonance imaging (rs-fMRI). Both patients with IGD and ibGD were started on bupropion SR 150 mg/day, which was then increased to 300 mg/day. The decision to adjust the dose was made by a psychiatrist (Doug Hyun Han) at the second-week visit on the basis of tolerability and efficacy. At the end of 12 weeks of bupropion treatment, clinical scales and rs-fMRI scans were repeated in all participants (Figure 1). The Chung-Ang University Hospital Institutional Review Board approved the research protocol for this study, and written informed consent was provided by all participants.

### MRI Acquisition and Preprocessing

Brain FC in the resting state was assessed using 3 T blood-oxygen-level dependent functional MRI (Philips Achieva 3.0 T TX MRI scanner; TR = 3 s; scan period, 12 min; 240 volumes; 128 × 128 matrix; 40 slices at a 4.0-mm slice thickness). Preprocessing consisted of despiking (AFNI: 3dDespike), motion correction (SPM 12b), coregistration to Magnetization Prepared RApid Gradient Echo image (SPM 12b), normalization to MNI space (SPM 12b), temporal detrend (Matlab: detrend.m), bandpass filtering (Matlab: idealfilter.m), and voxelwise regression of identically bandpass filtered time series of six head motion parameters (realignment steps with six rigid-body parameters characterizing the estimated subject motion for each subject), degraded cerebrospinal fluid, degraded white matter, and facial soft tissues (Matlab) as previously described (31). To address the possibility of micro-head movements affecting connectivity results (32), censoring of time points with a head motion >0.2 mm was performed, but no regression of the global signal was performed (31).

We extracted 12 regions of two brain networks [four from the DMN: mPFC, right/left lateral parietal cortex (LPRt/LPLt), and PCC; eight from the CCN: right/left DLPFC (DLPFCRt/DLPFCLt), right/left inferior PFC (IFGRt/IFGLt), right/left posterior parietal cortex (PPCRt/PPCLt), and right/left presupplementary motor area] from the AAL atlas of the brain (networks.nii/.txt/.info). Using the CONN-fMRI functional connectivity toolbox (ver.15; www.Nitrc.org/projects/conn), Fisher-transformed correlation coefficients were calculated for each pair of regions of interest in each subject. Between-group effects were considered significant with a cluster-level false discovery rate (FDR)q < 0.05, considering the multiple comparison correction over the correction of 66 pairs of 12 regions.

### Statistics

Demographic and clinical characteristics of IGD, ibGD, and healthy comparison subjects were analyzed using analysis of variance (ANOVA) tests with statistical significance set at *p* < 0.05. The correlations between clinical scales and brain connectivity were assessed using Spearman correlation with statistical significance set at *p* < 0.05. All statistical assessments were performed using SPSS 18.0 (SPSS Inc., Chicago, IL, USA).

## Results

### Changes in Clinical Symptoms After 12 Weeks of Bupropion Treatment

At baseline, there were no significant differences in age, education years, and IQ between IGD patients, ibGD patients, and healthy comparison subjects. However, there were significant differences in BISBAS (*F* = 6.56, *p* < 0.01), BDI (*F* = 4.68, *p* = 0.02), K-ARS (*F* = 24.09, *p* < 0.01), YIAS (*F* = 70.94, *p* < 0.01), and YBOCS-PG (*F* = 82.68, *p* < 0.01) scores between the three groups. The *post hoc* test showed no significant differences in BDI, K-ARS, and BISBAS scores between the IGD and ibGD groups. The YIAS scores in the IGD group were higher than those in the ibGD group (*z* = 4.58, *p* < 0.01) while the YBOCS-PG scores in the ibGD group were higher than those in the IGD group (*z* = 4.60, *p* < 0.01) (Table 1).

**Table 1:** Demographic and clinical characteristics.

	IGD		ibGD		HC
			
	Baseline	Follow-up	Baseline	Follow-up	
Age	25.3 ± 5.2		25.0 ± 4.9		25.7 ± 4.7
Education year	12.8 ± 2.6		12.1 ± 2.5		13.1 ± 2.3
IQ	99.0 ± 12.5		97.7 ± 15.3		103.8 ± 9.9
Alcohol (yes/no)	10/6		10/5		12/3
Smoking (yes/no)	8/8		9/6		8/7
BDI	9.7 ± 56.2	5.7 ± 2.8	14.1 ± 8.3	9.4 ± 3.4	6.1 ± 4.2
K-ARS	13.0 ± 4.5	9.3 ± 3.1	18.8 ± 7.7	14.4 ± 4.9	5.4 ± 3.4
BISBAS	47.6 ± 4.9	47.6 ± 4.9	50.7 ± 6.0	50.7 ± 6.0	49.0 ± 8.1
YIAS	68.9 ± 8.8	54.8 ± 8.2	38.3 ± 9.0	36.5 ± 7.4	37.6 ± 6.6
YBOCS-PG	5.7 ± 2.2	5.1 ± 1.8	17.8 ± 4.6	12.2 ± 4.3	4.1 ± 1.8

*IGD, Internet gaming disorder; ibGD, Internet-based gaming disorder; HC, healthy comparison subjects; IQ, intelligence quotient; BDI, Beck Depression Inventory; K-ARS, Korean ADHD Rating Scale; BISBAS, Behavioral Inhibitory System Behavioral Activation System; YIAS, Young Internet Addiction Scale; YBOCS-PG, Yale-Brown Obsessive Compulsive Scale for pathologic gambling*.

After the 12-week bupropion treatment, the BDI (*z* = −2.68, *p* < 0.01), K-ARS (*z* = −2.81, *p* < 0.01), BISBAS (*z* = −2.81, *p* < 0.01), and YIAS (*z* = −2.81, *p* < 0.01) scores improved in the IGD group while the BDI (*z* = −2.09, *p* = 0.04), K-ARS (*z* = −2.81, *p* < 0.01), BISBAS (*z* = −2.81, *p* < 0.01), and YBOCS-PG (*z* = −2.80, *p* < 0.01) scores improved in the ibGD group. However, there were no significant intergroup differences with regards to changes in the clinical scales during the 12-week period (Table 1).

### Changes in Brain FC After 12 Weeks of Bupropion Treatment

**Figure 2:**
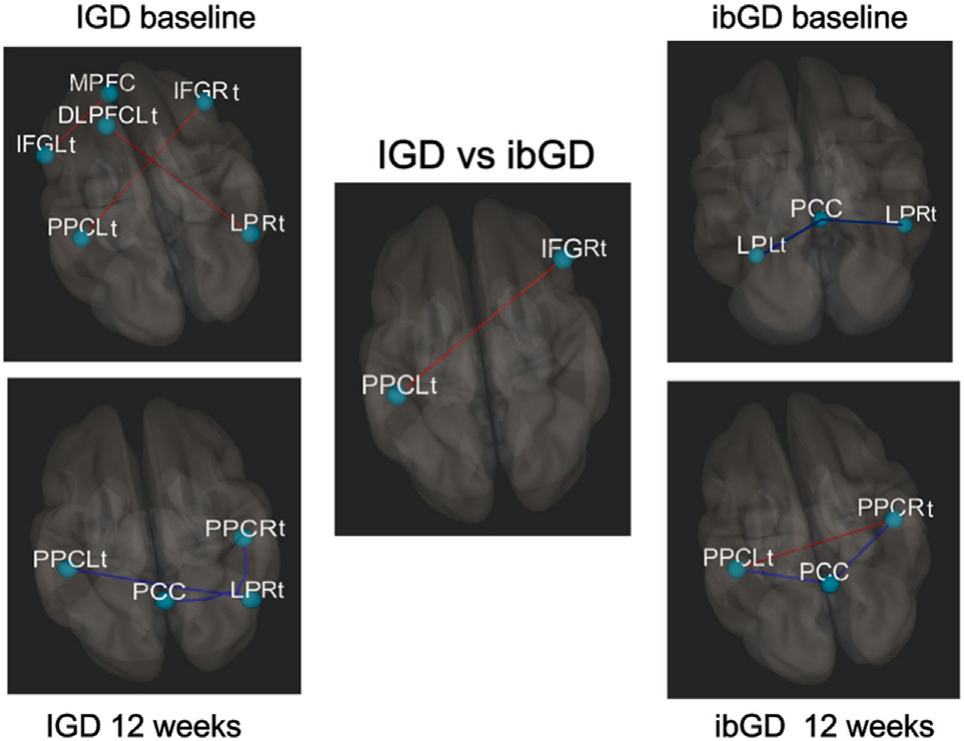
Changes in brain functional connectivity after 12 weeks of bupropion treatment. Red line: increased functional connectivity (FC), blue line: decreased FC, In the IGD group at baseline, the functional correlation between the middle frontal gyrus (MPFC) and left inferior prefrontal cortex (IFGLt) (*t* = 3.39, FDRq = 0.0026), left dorsolateral prefrontal cortex (DLPFCLt) and right lateral parietal cortex (LPRt) (*t* = 3.34, FDRq = 0.0030), and left posterior parietal cortex (PPCLt) and IFGRt (*t* = 3.67, FDRq = 0.0013). At 12 weeks, the functional correlation between the posterior cingulate cortex (PCC) and LPRt (*t* = −3.26, FDRq = 0.0017), LPRt and PPCRt (*t* = −3.16, FDRq = 0.0023), and LPRt and PPCLt (*t* = −3.42, FDRq = 0.0012). In ibGD group at baseline, the functional correlation between the PCC and LPLt (*t* = −3.36, FDRq = 0.0014), PCC and LPRt (*t* = −3.26, FDRq = 0.0027). At 12 weeks, the functional correlation between the PCC and PPCLt (*t* = −3.23, FDRq = 0.0031), PCC and PPCRt (*t* = −3.25, FDRq = 0.0031). The functional correlation between the PPCLt and PPCRt (*t* = 3.12, FDRq = 0.0042). In the IGD vs ibGD comparison (repeated measure analysis of variance), the ibGD group showed increased FC between IFGRt and PPCLt (*F* = 3.67, *p* = 0.0013), compared with IGD group.

In the IGD group at baseline, the FC between the MPFC and IFGLt (*t* = 3.39, FDRq = 0.0026), DLPFCLt and LPRt (*t* = 3.34, FDRq = 0.0030), and PPCLt and IFGRt (*t* = 3.67, FDRq = 0.0013) was higher than that in the healthy subjects. After 12 weeks of bupropion treatment, the FC between the PCC and LPRt (*t* = −3.26, FDRq = 0.0017), LPRt and PPCRt (*t* = −3.16, FDRq = 0.0023), and LPRt and PPCLt (*t* = −3.42, FDRq = 0.0012) were lower than baseline (Figure 2).

In the ibGD group at baseline, the FC between the PCC and LPLt (*t* = −3.36, FDRq = 0.0014) as well as PCC and LPRt (*t* = −3.26, FDRq = 0.0027) was lower than that in healthy subjects. After 12 weeks of bupropion treatment, the FC between the PCC and PPCLt (*t* = −3.23, FDRq = 0.0031) as well as the PCC and PPCRt (*t* = −3.25, FDRq = 0.0031) was decreased while that between PPCLt and PPCRt (*t* = 3.12, FDRq = 0.0042) had increased compared with baseline (Figure 2).

A repeated measures ANOVA revealed that the ibGD group showed increased FC between IFGRt and PPCLt (*F* = 3.67, *p* = 0.0013), compared with the IGD group (Figure 2).

### Correlation Between the Changes in Clinical Scales and the Changes in Brain FC

**Figure 3:**
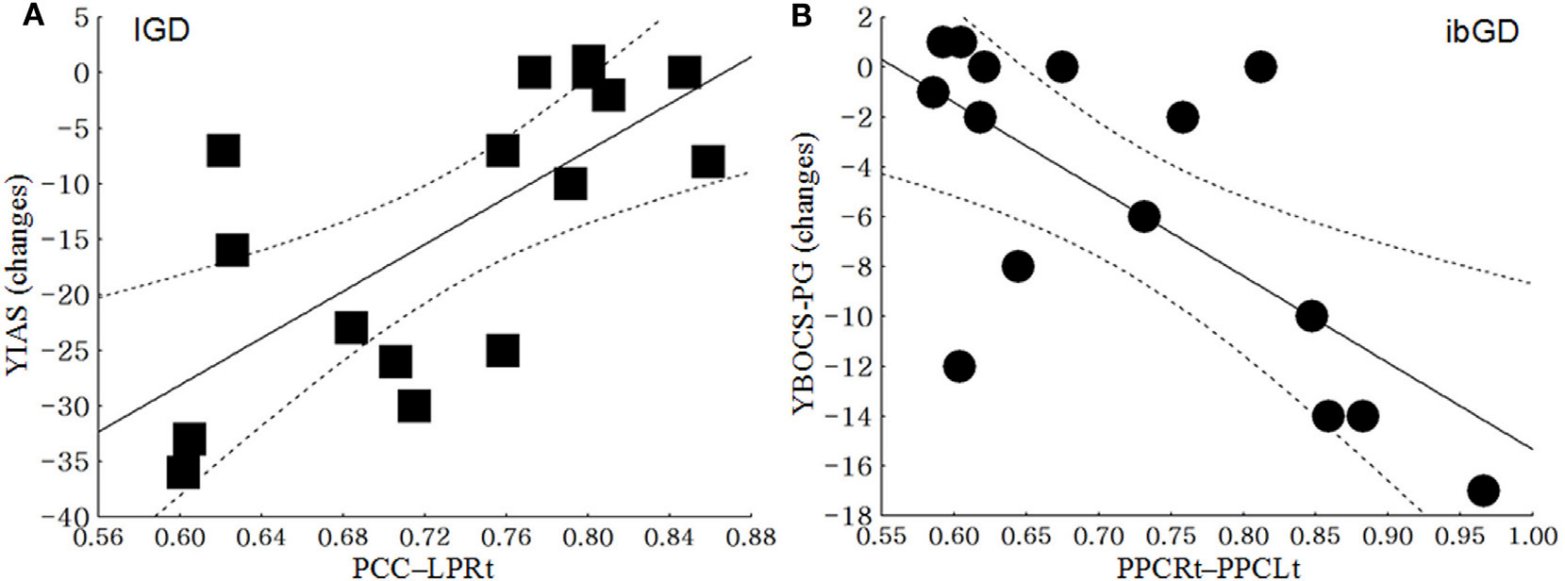
Correlation between the changes in clinical scales and the changes in brain functional connectivity. **(A)** In the Internet gaming disorder (IGD) group, the functional connectivity between the posterior cingulate cortex (PCC) and right lateral parietal cortex (LPRt) was positively correlated with the changes in the Young Internet Addiction Scale scores from baseline to 12 weeks (*r* = 0.69, *p* < 0.01). **(B)** In the ibGD group, the changes in FC between the left posterior parietal cortex (PPCLt) and right posterior parietal cortex (PPCRt) were negatively correlated with the changes in the Yale-Brown Obsessive Compulsive Scale for pathologic gambling (YBOCS-PG) scores from baseline to 12 weeks (*r* = −0.68, *p* < 0.01).

In the IGD group, the functional correlation between PCC and LPRt was positively correlated with changes in YIAS scores from baseline to 12 weeks (*r* = 0.69, *p* < 0.01). In the ibGD group, the changes in FC between the PPCLt and PPCRt were negatively correlated with the changes in YBOCS-PG scores from baseline to 12 weeks (*r* = −0.68, *p* < 0.01) (Figure 3).

## Discussion

### Changes in Clinical Symptoms in Response to Bupropion Treatment

In this study, 12-week bupropion treatment improved the severity of IGD and ibGD as well as the associated clinical symptoms in both patient groups. The effectiveness of bupropion for the treatment for IGD has been reported in previous studies (8, 16). Twelve weeks of bupropion treatment has been shown to reduce the severity of IGD as well as depressive symptoms in IGD patients with major depressive disorder (8). In a comparison of escitalopram and bupropion treatment, bupropion showed greater effectiveness in improving impulsivity and attention (12). The effectiveness of bupropion in patients with GD is a matter of debate (5, 6). Although Black et al. (5) reported the effectiveness and tolerability of bupropion in patients with GD, its effectiveness in GD symptom reduction was not greater than that of placebo (5). However, Dannon et al. (6) declared that bupropion was as effective as naltrexone in patients with GD (6). Due to the dual action of bupropion with regards to the inhibition of norepinephrine and dopamine reuptake, it is thought to be effective for reducing impulsive behaviors in both IGD and ibGD patients (33, 34). Impulsivity is a well-known correlate of prototypical behavioral addictions with steep discounting of delayed rewards (35). This steep discounting of delayed rewards is associated with the dopamine-based neuromodulatory system (36).

### Changes in Brain FC After 12 Weeks of Bupropion Treatment

In response to 12 weeks of bupropion treatment, the FC within the DMN as well as that between the DMN and CCN decreased in the IGD group, while the FC within the CCN increased in the ibGD group. The IGD and ibGD groups showed different brain FC patterns in response to bupropion treatment. In the IGD group, the FC within the posterior DMN as well as the FC between the DMN and CCN decreased after the 12-week treatment period. Moreover, the FC between the PCC and LPRt in the IGD group was positively correlated with changes in YIAS after the 12-week bupropion treatment period. These results were consistent with our previous study showing decreased FC within the DMN and between the DMN and the salience network (12). Decreased FC within the DMN may be associated with increased norepinephrine and dopamine, as observed in the DMN in response to the administration of atomoxetine (37). The dual action of bupropion in increasing norepinephrine and dopamine signaling is similar to the mechanism of action of modafinil (38). The increased FC within the DMN was thought to be related to impulsivity, risky decision-making, and attention deficits (17, 39). Therefore, decreasing the FC within the DMN and the FC between the DMN and other networks may reduce impulsive behavior, such as excessive Internet game-playing or gambling.

In the ibGD group, the FC within the posterior DMN decreased while that within the CCN increased after the 12-week bupropion treatment period. Moreover, the FC within the CCN (IFGRt – PPCLt) in the ibGD group was much higher than that in the IGD group. The FC within the CCN (PPCLt − PPCRt) in the IGD group was negatively correlated with changes in the YBOCS-PG scores after the 12-week bupropion treatment period. The failure of self-regulation in patients with GD is thought to occur due to failure in prefrontal-mediated top-down inhibitory control (40). The top-down circuitry is reported to be associated with decision errors (36) as well as dopamine transmission (41). In addition, areas of the fronto-parietal cortices are engaged in top-down attention and cognitive control (42). Therefore, the pharmacodynamic activity of bupropion (dopamine stimulation) may enhance the CCN (fronto-parietal areas) by promoting activity within the top-down circuitry in patients with ibGD. Taken together, IGD and ibGD appear to share similar characteristics of decreased impulsivity and decreased FC within the DMN after bupropion treatment. However, bupropion was more effective at increasing the FC within the CCN, which is associated with the correction of decision errors.

### Limitations

There were several limitations in this study. First, the small number of subjects limits the generalizability of the results. Due to the small number of subjects, only two brain networks of interest were used to compare the FC changes between the two groups in response to bupropion treatment. Second, as this study did not have a placebo control group, we cannot rule out the possibility that we were seeing a placebo effect. Finally, because the healthy control subjects did not participate in follow-up assessments, we did not have a measure of test-retest variability. Future studies should include a larger number of subjects as well as follow-up information for healthy control subjects.

## Conclusion

Bupropion shows promise for improving problematic behaviors in both IGD and ibGD. However, the pharmacodynamics of bupropion differed between the two groups, whereby the FC within the DMN as well as between the DMN and CCN decreased in patients with IGD, whereas the FC within the CCN increased in patients with ibGD after 12 weeks of bupropion treatment.

## Ethics Statement

The Chung-Ang University Hospital Institutional Review Board approved the research protocol for this study, and written informed consent was provided by all participants.

## Author Contributions

JH, SK, and DH contributed to patients’ recruitment, data collection, and processing. SB, JH, and DH analyzed the data. All the authors participated to drawing up the manuscript, were involved to the intellectual workup for the article, and read and approved the final manuscript.

## Conflict of Interest Statement

No competing personal, professional, or financial interests exist.
